# LeadMine: a grammar and dictionary driven approach to entity recognition

**DOI:** 10.1186/1758-2946-7-S1-S5

**Published:** 2015-01-19

**Authors:** Daniel M Lowe, Roger A Sayle

**Affiliations:** 1 NextMove Software Ltd, Innovation Centre, Unit 23, Science Park, Milton Road, Cambridge, UK

**Keywords:** LeadMine, grammars, dictionaries, chemical entity recognition, CHEMDNER, Biocreative IV

## Abstract

**Background:**

Chemical entity recognition has traditionally been performed by machine learning approaches. Here we describe an approach using grammars and dictionaries. This approach has the advantage that the entities found can be directly related to a given grammar or dictionary, which allows the type of an entity to be known and, if an entity is misannotated, indicates which resource should be corrected. As recognition is driven by what is expected, if spelling errors occur, they can be corrected. Correcting such errors is highly useful when attempting to lookup an entity in a database or, in the case of chemical names, converting them to structures.

**Results:**

Our system uses a mixture of expertly curated grammars and dictionaries, as well as dictionaries automatically derived from public resources. We show that the heuristics developed to filter our dictionary of trivial chemical names (from PubChem) yields a better performing dictionary than the previously published Jochem dictionary. Our final system performs post-processing steps to modify the boundaries of entities and to detect abbreviations. These steps are shown to significantly improve performance (2.6% and 4.0% F_1_-score respectively). Our complete system, with incremental post-BioCreative workshop improvements, achieves 89.9% precision and 85.4% recall (87.6% F_1_-score) on the CHEMDNER test set.

**Conclusions:**

Grammar and dictionary approaches can produce results at least as good as the current state of the art in machine learning approaches. While machine learning approaches are commonly thought of as "black box" systems, our approach directly links the output entities to the input dictionaries and grammars. Our approach also allows correction of errors in detected entities, which can assist with entity resolution.

## Background

With the rapidly increasing volume of scientific publications machine assisted knowledge extraction is becoming a necessity. Entity recognition allows the association of concepts with a document such as a research article, patent or thesis. Knowledge of the position of entities within text facilitates higher level relationship extraction (e.g. associating quantities with chemical entities), identifying interactions between chemicals and other entity types (e.g. chemical-protein), determining the role of chemicals in a chemical reaction etc. All such activities work best when the chemical entity recognition step has both high recall and precision.

To test the current state of the art in chemical entity recognition, and drive further innovation in the field, BioCreative IV introduced the Chemical compound and drug name recognition task (CHEMDNER) [1]. This task consisted of a corpus of 10,000 PubMed abstracts that were annotated by domain experts to provide a gold standard. 3,500 of these were released to task participants as the "training corpus" with a further 3,500 released as the "development corpus". These 7,000 abstracts were used by participants to train and validate their solutions. The remaining 3,000 abstracts formed the test set and were provided to participants (along with 17,000 decoy abstracts) so that they could predict the entities that were annotated by the domain experts.

Attempts to tackle the problem of chemical entity recognition have invariably identified that the problem is not amenable to pure dictionary approaches due to the continuing discovery of novel compounds and the many ways in which systematic nomenclature allows compounds to be named [2]. Hence, state of the art systems use machine learning techniques to learn weights for pre-engineered features indicative of chemical nomenclature, e.g. character sequences, word morphology, features of surrounding words etc. Examples include OSCAR4 which employs a maximum-entropy Markov model [3], and ChemSpot which employs a conditional random field model [4]. Comprehensive reviews of the area have been performed by Vazquez et al. [5] and Gurulingappa et al. [6].

LeadMine instead encodes the rules used to describe systematic chemical nomenclature (as grammars), with large dictionaries being used for trivial names (unsystematic names). Compared to machine learning approaches this makes the results easily understandable; false positives can be pin-pointed to a particular grammar/dictionary and false negatives are readily corrected by adding the relevant nomenclature rule to a grammar or trivial name to a dictionary.

## Implementation

**Figure 1:**
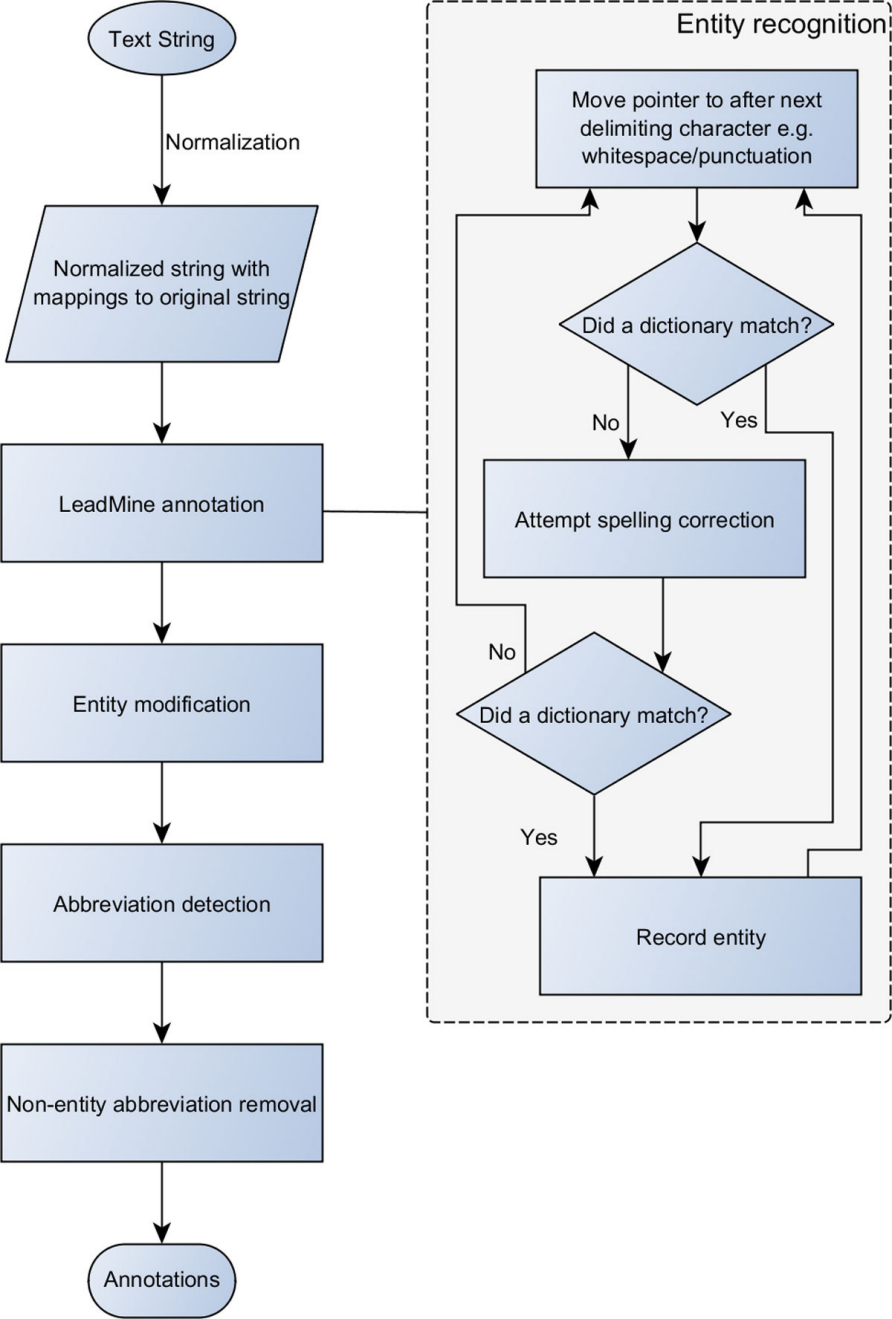
**Annotation workflow diagram**.

Figure 1 shows the workflow we developed; the steps are explained below. It should be noted that every step subsequent to LeadMine annotation can be considered a form of post-processing, and any or all of these steps may be omitted.

### Normalization

A normalization step is performed to address the issue of there being many Unicode characters with similar meaning. For example `(backtick), ‘ and ’ (single quotation marks) and ′ (prime) are all converted to apostrophe. Another example is œ which is converted to oe. This step reduces the number of superficial variants that dictionaries/grammars need to match. The indexes of characters in the original string are associated with indexes in the normalized string to allow mapping back to the original input.

The normalization step also facilitates processing of XML documents by removing stylistic tags and considering all other tags as delimiting paragraphs. For example, <p>H<sub>2</sub>O</p> is normalized to H2O. The ability to handle XML input was, except in a handful of cases, irrelevant to PubMed abstracts.

### LeadMine annotation

The rules for chemical nomenclature are encoded as formal grammars, e.g.

alkaneStem : 'meth' | 'eth' | 'prop' …

alkane: alkaneStem 'ane'

**Figure 2:**
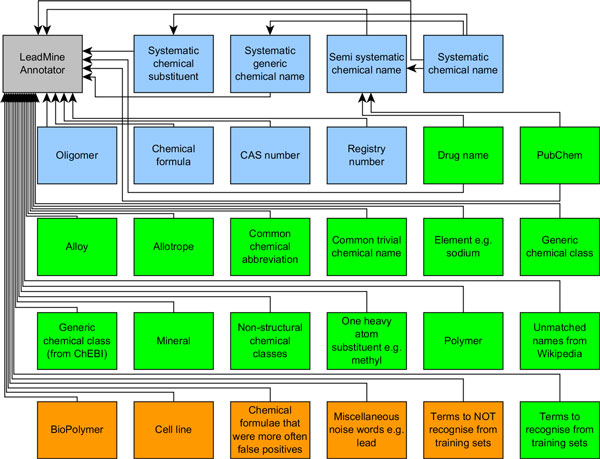
**Dictionaries employed by LeadMine for the CHEMDNER task**. (blue: grammars, green: traditional dictionaries, orange: blocking dictionaries)

Our grammar for systematic chemical names currently contains 486 of such rules. As shown in Figure 2, grammars may inherit rules from other grammars and may employ dictionaries as part of their rules. The semi-systematic chemical name grammar allows any number of systematic chemical substituents followed by a trivial name from the Drug name or PubChem dictionary.

To efficiently match against our grammars we convert them to finite-state machines [7].

For example:

Digit1to9 : '1' | '2' |'4' |'5' |'6' |'7' |'8' |'9'

Digit : Digit1to9 | '0'

Cid : 'CID:' Digit1to9 Digit*

**Figure 3:**
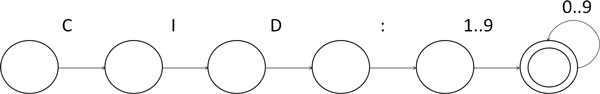
**State machine for a PubChem compound identifier**. Any string which, once consumed by the state machine, leaves the state machine in the double circled state is accepted.

Can be converted to the state machine shown in Figure 3.

The state machine representation restricts us to the subset of rules that may be expressed by a regular grammar, e.g. a rule may not reference itself. As correct nesting of brackets is not generally possible with this constraint, while matching characters against a dictionary a record of the brackets seen is kept. A valid dictionary match is required to have well-nested balanced brackets.

Due to the complexity of IUPAC nomenclature it is impractical to build a single state machine that covers all IUPAC nomenclature while still rejecting syntactically invalid chemical names. Hence, our systematic chemical grammars are represented as a state machine in which the transitions, instead of corresponding to characters, correspond to other state machines (in which transitions do correspond to characters). This separation allows the main state machine to keep track of overall state while the other state machines match actual text. For example, which characters are allowed after seeing the substituent 'ethyl' differs between 'ethylbenzene' and 'acetic acid ethyl ester'. By storing this distinction in the main state machine we can use the same state machine to match 'ethyl' in both cases, despite the difference in context.

In addition to systematic chemical names we also employed grammars for the entities in Table 1. The grammars with subtypes are structured by combining the subtypes to form the ultimate grammar; hence it would be trivial to just match entities corresponding to the one of the subtypes if the distinction were considered important.

**Table 1:** Other chemical entity types recognized by LeadMine using grammars.

Grammar name	Subtype	Example
Chemical formula	Sum formula	C_20_H_25_NO_6_

..	Line formula (complete molecule)	CH3CH2CH2Cl

..	Line formula (linker)	CH2CH2

..	Line formula (substituent)	CH3CH2

..	Salt	MgSO_4_

Oligomer	Peptide	Cys-Tyr-Phe-Gln-Asn-Cys-Pro-Arg-Gly-NH2

..	Oligosaccharide	α-L-Fuc*p*-(1→4)-[β-D-Gal*p*-(1→3)]-β-D-Glc*p*NAc-(1→3)-β-D-Gal*p*-(1→4)-D-Glc-ol

..	Oligonucleotide	3'-AATG-5'

CAS Number	n/a	2634-33-5

Registry Number	n/a	GSK2248761

The PubChem dictionary is our primary source of trivial names. It contains 1.48 million terms. It was produced by running a series of filters against the unfiltered list of synonyms provided by PubChem [8] (~100 million terms). As most synonyms in PubChem are database identifiers which are either not useful for text mining or are better expressed by grammar, the number of useful synonyms is far smaller.

Previous work in the area of chemical dictionary preparation by Hettne et al. [9] indicated PubChem to be the chemical database whose synonyms had highest recall. However the use of synonyms from this database was hampered by such poor precision that it was not used in their final ensemble dictionary (Jochem). To address this we developed an extensive series of filters to remove ambiguous (could be a chemical name but typically was not) and incorrect (protein names, non-chemical English words etc.) synonyms without significantly lowering the recall. A comparison between Jochem and our dictionary is discussed later.

The following filters and term expansion rules were applied:

• Reject if the associated structure was determined to be a tetrasaccharide (or longer) or hexadecapeptide (or longer). This conformed to the letter of the CHEMDNER annotation guidelines.

• Reject if contains unbalanced brackets

• Remove superfluous bracketed qualifiers, e.g. '(INN)'. Reject if the qualifier is unknown.

• Reject if preceded/followed by a qualifier, e.g. 'derivative', 'analog', 'solution'

• Reject if ≤ 3 characters

• Reject if starts or ends with a hyphen

• Reject if contains a comma followed by a space

• Reject if matched by another LeadMine dictionary

• Reject if is a known dubious synonym, e.g. 'AstraZeneca'

• Reject if the first word is a non-chemical English word unless the next word is 'acid'

• Reject if contains a dubious word, e.g. 'gene', 'inhibitor'

• Reject if the name is a depositor's external name, i.e. it is a catalogue number

• Reject if a regular expression for common catalogue numbers matches

• Reject if contains any of the following characters ? ! \ | % @ ;

• Reject if is a protein data bank (PDB) code

• Reject if is an InChI key

• If contains Greek letters written out in Latin characters, e.g. 'alpha-' generate a variant that instead uses the Greek character, e.g. 'α-'

• If the last word consists of digits, generate variants where the preceding space is deleted or replaced by a hyphen, e.g. 'KF 17837' will also give 'KF-17837' and 'KF17837'

• If the last word is a letter or letter followed by a digit, generate variants where the preceding space is replaced by a hyphen, e.g. 'Bisphenol A' will also give 'Bisphenol-A'

In addition we employed the dictionaries shown in Table 2.

**Table 2:** Additional chemical dictionaries used by LeadMine.

Dictionary	Number of terms	Example	Construction methodology
Alloy	206	pig iron	Manually constructed

Allotrope	72	red phosphorus	Manually constructed

Common chemical abbreviation	224	TMEDA	Manually constructed

Common trivial chemical name	574	adam's catalyst	Manually constructed

Drug name	11397	vancomycin	Manually constructed

Element	227	protactinium	Manually constructed

Generic chemical class	2254	quaternary amine	Manually constructed

Generic chemical class from ChEBI	3917	keto steroids	Derived from ChEBI [10] terms that are referenced in an "is a" relationship by another term

Mineral	5100	paragonite	International Mineralogical Association names with some manual additions

Non-structural chemical classes	18	bronsted lowry acid	Manually constructed. Terms that do not strictly convey structural information but were nonetheless annotated in the corpus

One heavy atom substituent	11	methyl	Manually constructed

Polymer	531	polyethylene glycol 8000	Manually constructed. Biochemical polymers blocked by another dictionary

Wikipedia	171	beefy meaty peptide	Terms from Wikipedia chemboxes not matched by our other dictionaries

None of the aforementioned dictionaries or grammars directly take advantage of the annotated corpus. Hence, we used the system's false negatives to derive a dictionary of terms to include (Include list) and the system's false positives to derive a dictionary of terms to exclude (Stop list). When evaluating our final system on the test set we used the union of the training and development sets to derive these dictionaries. Candidate terms were only included if they increased f-score on the combined training and development set (some false negative terms are highly ambiguous and their inclusion reduced precision more than the increase in recall).

The CHEMDNER annotation guidelines indicate that biopolymers should not be annotated but that synthetic polymers should be. As LeadMine's polymer dictionary does not make this distinction, we determined which of the entries in the dictionary were natural polymers and use these to produce a dictionary of terms to exclude. This distinction can become quite unclear, e.g. is an artificial derivative of a natural polymer synthetic?

While most chemical names can be determined to be chemical names without context, this is not the case for short chemical formulae. Obvious counter examples include 'In' (indium) and 'As' (arsenic). As such terms are usually of lesser importance anyway, we have not attempted to disambiguate their meaning from the context of their usage. Instead, we have simply used the training/development set corpora to derive which chemical formulae found by our system are more often false positives. The results of this were used as a dictionary of terms to exclude.

### Spelling correction

LeadMine supports performing edit operations to correct a potential entity, such that it then corresponds to an entity recognized by a grammar/directory. These edits may be character insertions, deletions, substitutions or transpositions. This correction process is facilitated by a refined version of the algorithm described by Sayle et al. [7]. Common optical character recognition mistakes, typos (e.g. floro) and minor mistakes (e.g. erroneous space, comma instead of a period, missing hyphen) are assigned lower costs such that the algorithm may perform more of these correction operations. In the case that the algorithm suggests multiple corrections that yield recognized entities, the cost of the corrections is used to choose which is preferred.

Spelling correction may be customized on a per dictionary/grammar basis as to whether correction should be performed, how many unparameterized edits may be performed and how long an entity must be for spelling correction to be allowed. As PubMed abstracts generally have few actual errors, we restricted corrections to just the parameterized errors and enabled it on dictionaries that matched chemical name-like entities.

### Entity modification

PubMed abstracts frequently contain novel trivial and semi systematic terms. This contrasts with patents (for which LeadMine was originally developed) where fully systematic names are more frequently used. Grammar based approaches work when the entities to be recognized are systematic in nature, while dictionaries cannot recognize novel terms.

Fortunately, in chemistry, many novel terms arise from combination or derivatization of known terms. For example complicated chemical family names, e.g. 'monoterpene pyridine alkaloids' may not appear in any lexicon but the individual components will. This example will be recognized as three distinct entities, which can then by an entity merging step be combined to give the entire entity. In other cases part of an entity may be recognized, e.g. in '(S)-nornicotine' nornicotine will be recognized and entity extension can then infer that the rest of the word is likely to be part of this entity.

Our entity modification process comprises the following steps:

#### 1. Entity extension

Entities are extended until one of the following is reached: whitespace, a mismatched bracket or a non-chemical English word/noise word. Additionally if an entity was entirely enclosed in balanced brackets and entity extension starting from before and/or after the brackets yielded a longer entity we used these entity boundaries.

An exception was made for the case of two entities separated by a hyphen where both corresponded to specific compounds. In this case the end and start of the entities respectively are not extended and the entities are not merged. Such a construct often indicates a mixture, e.g. 'Resorcinol-Formaldehyde'.

#### 2. Entity trimming

In accordance with the annotation guidelines, the start and end of entities are trimmed of "Non-essential parts of the chemical entity and name modifiers", e.g. 'group', 'colloidal', 'dye' etc.

#### 3. Entity merging

Entities that overlap after entity extension are merged together. Entities that are space separated are merged together unless one of the entities is found to be an instance of the other entity. For example genistein isoflavonoid is not merged as genistein IS an isoflavonoid. These relationships are derived from the ChEBI [10] ontology.

#### 4. Removal of stop words

The aforementioned entity trimming process is repeated. If after trimming an entity corresponds to a stop term it is excluded. An example is 'gold nanoparticles' where 'nanoparticles' is excluded by trimming and 'gold' is explicitly not to be annotated according to the annotation guidelines.

#### 5. S-transferase special case

The 'S' in glutathione-S-transferase is annotated using its proximity to the glutathione entity.

### Abbreviation detection

We used an adapted version of the Hearst and Schwartz algorithm [11] to identify abbreviations of entities found by our system. By providing the "long form" (unabbreviated form) we avoid one of the issues with the algorithm, which is that it may not identify the complete unabbreviated form. We extended the algorithm to recognize abbreviations of the following forms:

• Tetrahydrofuran (THF)

• THF (tetrahydrofuran)

• Tetrahydrofuran (THF;

• Tetrahydrofuran (THF,

• (tetrahydrofuran, THF)

• THF = tetrahydrofuran

Abbreviations may contain brackets so long as they are balanced. The conditions described by Hearst and Schwartz are applied with the additional requirements that the short form must not be a common chemical identifier, e.g. '1a' or Roman numeral, e.g. 'II'. The minimum length of abbreviations is configurable and set to 3 for compliance with the annotation guidelines.

We also utilize a list of string equivalents to allow, for example, mercury to be abbreviated to Hg. Hence the algorithm knows that MeHg is a plausible abbreviation for methylmercury. Once an abbreviation has been detected all further instances of that string, in that particular document, are annotated.

### Non-entity abbreviation removal

In this step we postulate that an entity we have discovered is an abbreviation and use the Hearst and Schwartz algorithm to find a potential long form for it. If the algorithm finds a suitable long form and this long form is not also an entity or overlapping with an entity, we assume that the abbreviation entity is a false positive. We then remove it along with all other instances of it. For example when 'current good manufacturing practice (cGMP)' is seen, cGMP clearly doesn't mean cyclic guanosine monophosphate!

## Results

All data in this section relates to the evaluation of the ability to find chemical entity mentions in the CHEMDNER test set. Results were calculated using the evaluation script provided by the task organizers and the same metrics used by the organizers (micro-averaged F_1_-score) are reported.

## Discussion

### Post competition improvements

As our system already produced highly competitive results on the test corpus, improvements have been incremental in nature (Table 3). To keep the test corpus valid for testing, only the training/development sets were used to identify issues and performance improvements were validated against these sets. The following improvements each contributed about 0.1% f-score:

• PubChem dictionary regenerated from latest synonym data

• Improved precision of the PubChem dictionary by removing more ambiguous and incorrect synonyms

• Generated additional dictionary entries that use the actual Greek characters rather than Latin characters, e.g. 'α' instead of 'alpha'

• Turned on spelling correction on more of the dictionaries, e.g. PubChem

• Improved the precision of the Chemical Formula grammar (Table 1) by limiting sum formulae to just those with more than two digits, e.g. CDK2, CaCo, HeLa are no longer recognized as sum formulae.

• Fixed a few corner cases in entity extension, e.g. 'leucine-to-proline' is two entities

**Table 3:** Results on the CHEMDNER test set.

Configuration	Precision	Recall	F_1_-score
LeadMine competition submission (2013-10-07)	88.73%	85.06%	86.86%

Best competition submission	89.09%	85.75%	87.39%

LeadMine (2014-03-17)	89.90%	85.42%	87.60%

### Include lists and stop lists

**Figure 4:**
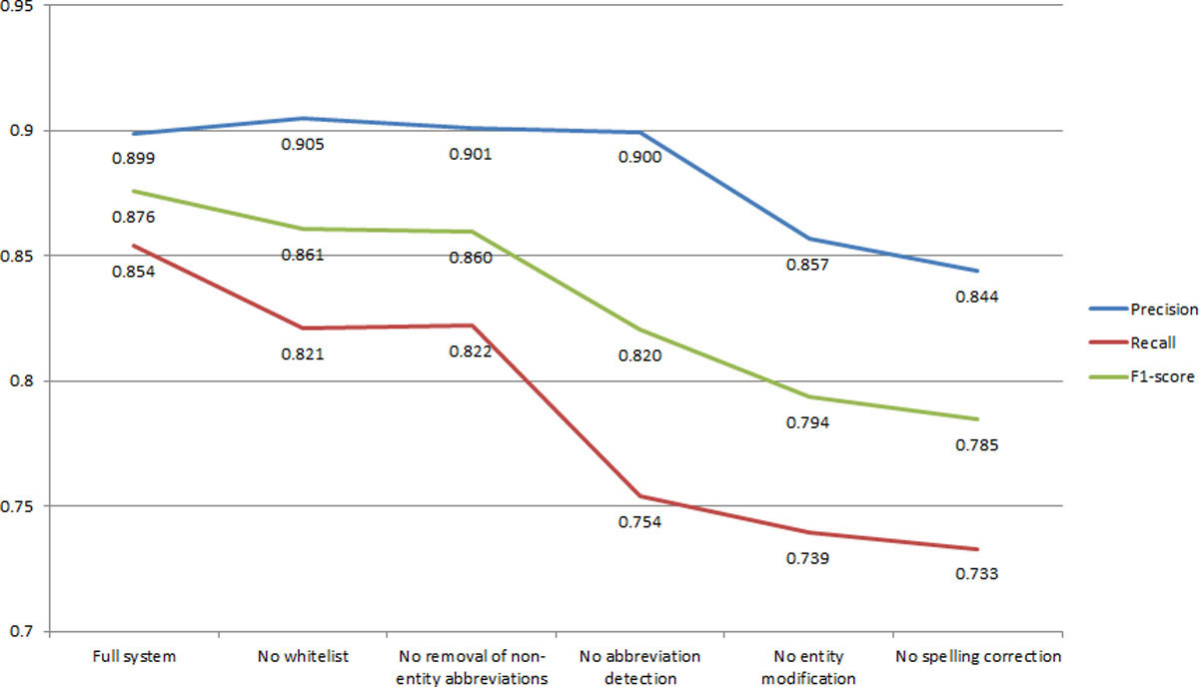
**Performance after incrementally removing each feature from the full system**.

The addition of an include list dictionary trained from the training/development corpus provided a significant increase in recall (Figure 4) with only a minimal loss of precision. This indicates that there are still gaps in the coverage of the system's dictionaries and grammars. This include list (currently containing 2969 terms) could be used to suggest areas where the system should be improved. Broadly the terms in the list fall into the following categories:

• Unusual trivial names, especially natural products, e.g. 'chikusetsusaponin L10', 'microgrewiapines A-C'

• Short, potentially ambiguous, terms, e.g. 'AP5', 'WB2', 'ZIP'

• Typographic errors, e.g. '(68) Ga(3+)' [erroneous space]

• Unusual chemical family names, e.g. '1,6-Diyne Carbonates and Esters', 'Antimony-Doped Tin Oxide', 'cyclic tri- and tetra-amine disulfides'

A dictionary derived from a database that specializes in natural products may be beneficial for the first of these categories.

Empirically it was found that the use of a stop list derived from the training/development corpus offered too little precision to offset the loss of recall [12], i.e. it reduced F_1_-score. This can be explained by the fact that most LeadMine false positives arise from getting part of an entity. This partial entity in a different context could be an actual entity so blocking the partial entity necessarily lowers recall. Hence our best performing solution just uses an include list.

### Speed

**Figure 5:**
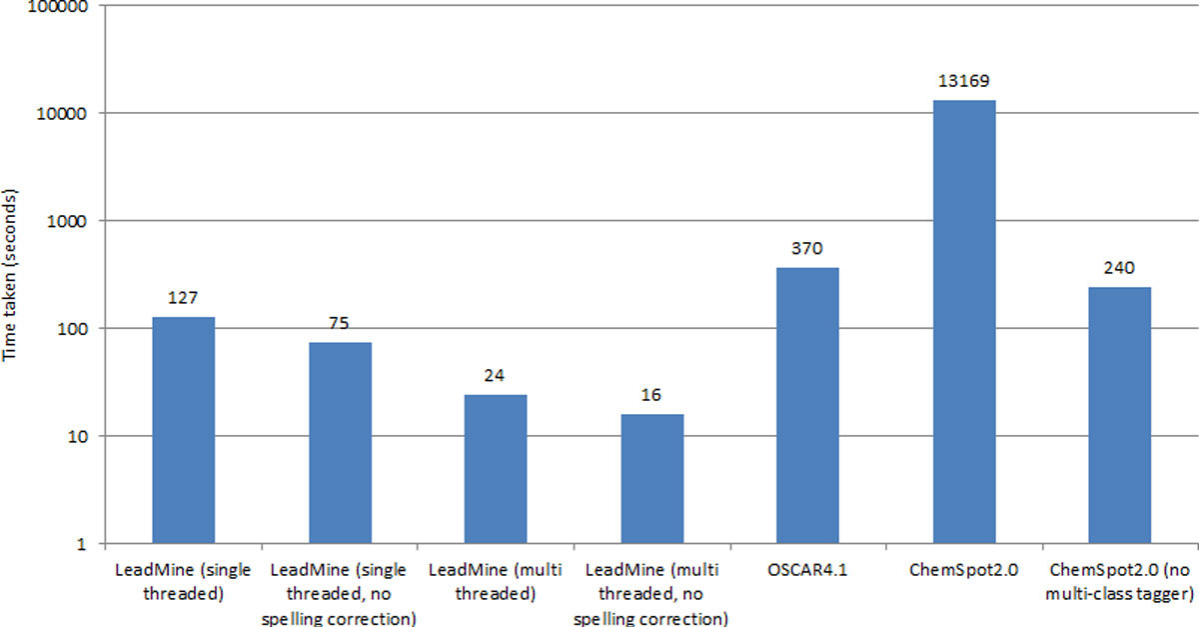
**Time to process the test set (20,000 PubMed abstracts) on an i7-3770k using Java 7u45 64-bit**. OSCAR4.1 and ChemSpot2.0 are shown to contrast with typical machine learning approaches. No entity normalization was performed and results are exclusive of initialization time (2 seconds, 4 seconds, 38 seconds and 23 seconds for LeadMine, OSCAR4.1, ChemSpot2.0 and ChemSpot2.0 without multi-class tagger, respectively)

As shown in Figure 5 our system is fast compared to other popular solutions such as OSCAR4 and Chemspot. LeadMine's speed is proportional to the number of dictionaries/grammars used (Figure 2). By keeping the dictionaries distinct LeadMine can determine the type of each entity found but further speed improvements could be achieved by merging dictionaries/grammars. The speed of matching primarily comes from our state machine based matching which minimizes the number of operations per character of the input document. This level of performance is useful for providing real-time annotation of full-text articles/patents. It also allows the processing of what may be considered "big data", e.g. the 3.5 million European Patent Office back-file, on a desktop machine in a little more than a day.

Memory usage also differs significantly between solutions, while OSCAR4.1 and LeadMine require less than 1GB, ChemSpot 2.0 requires either 15 or 9 GB depending on whether or not the multi-class tagger is used.

### Entity modification

Entity modification gave a significant improvement in both recall and precision (Figure 4) due to reducing the number of partial entity hits (which count as false positives) and increasing the number of hits with the correct entity boundaries. However, depending on the use case this increase in performance could be artificial; in all cases where entities have been modified after matching there is likely to be a significant increase in the difficulty of resolving these terms. For example nornicotine will be in PubChem and hence resolvable to a structure, but (S)-nornicotine will not. Similarly systematic chemical names, prior to modification, are likely to be resolvable by chemical name to structure algorithms.

### Trivial chemical name dictionaries

**Figure 6:**
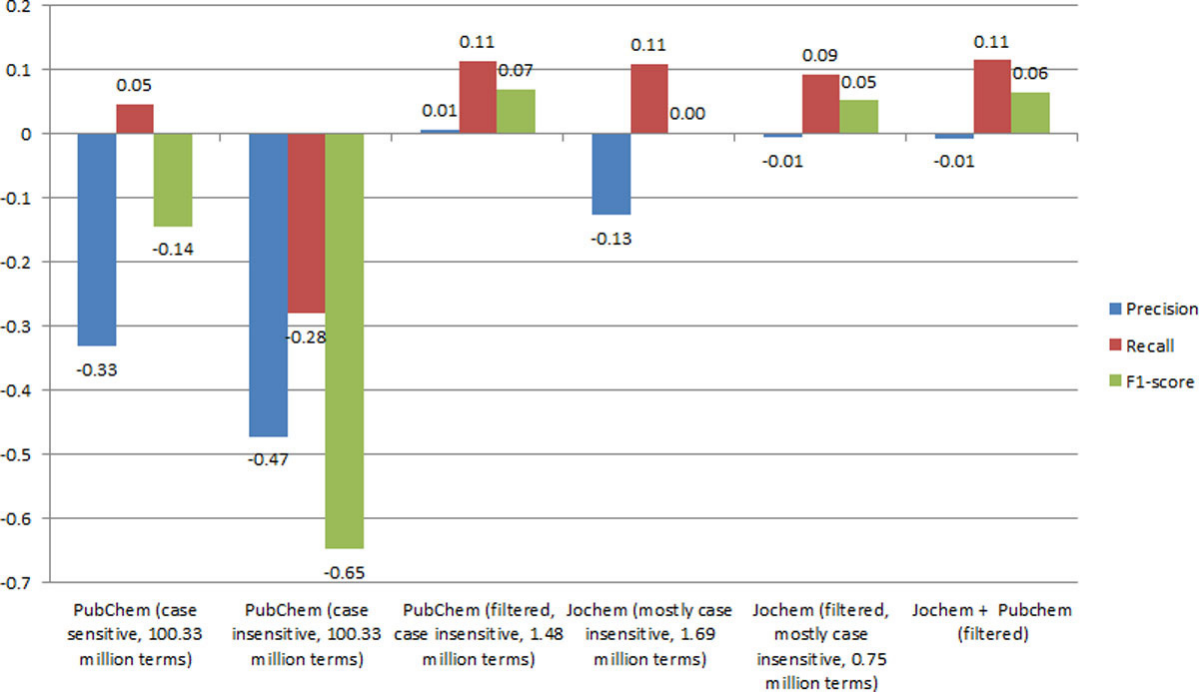
**Change in results by adding either a Jochem or PubChem dictionary to LeadMine configured with no include list or PubChem dictionaries**.

Figure 6 shows a comparison of the effects of different trivial name dictionaries on the system's performance. The abysmal performance of a case-insensitive PubChem dictionary is due to common English words, e.g. 'and' being matched. This is then compounded by the entity modification process merging these false positives with adjacent true positives such that the true positive is also lost, hence the loss in both precision and recall.

Jochem, out of the box, also possess a fair number of dubious terms (e.g. 'command', 'surpass', 'procure', 'optimizer') although it is overall a far cleaner resource than PubChem. Jochem's precision also benefited from the same filtering procedure we applied to PubChem although as structure information was not available for all terms we were unable to filter for long peptides/saccharides. Jochem contains indication of which terms should be treated case sensitively which we honored.

Jochem, both before and after filtering, has lower recall than our PubChem derived dictionary. Using the filtered PubChem and Jochem dictionaries in combination gave a negligible improvement in recall indicating that most of Jochem is a subset of PubChem. This is not entirely surprising as most of the databases that Jochem is aggregated from are PubChem depositors.

### Beyond chemistry

**Figure 7:**
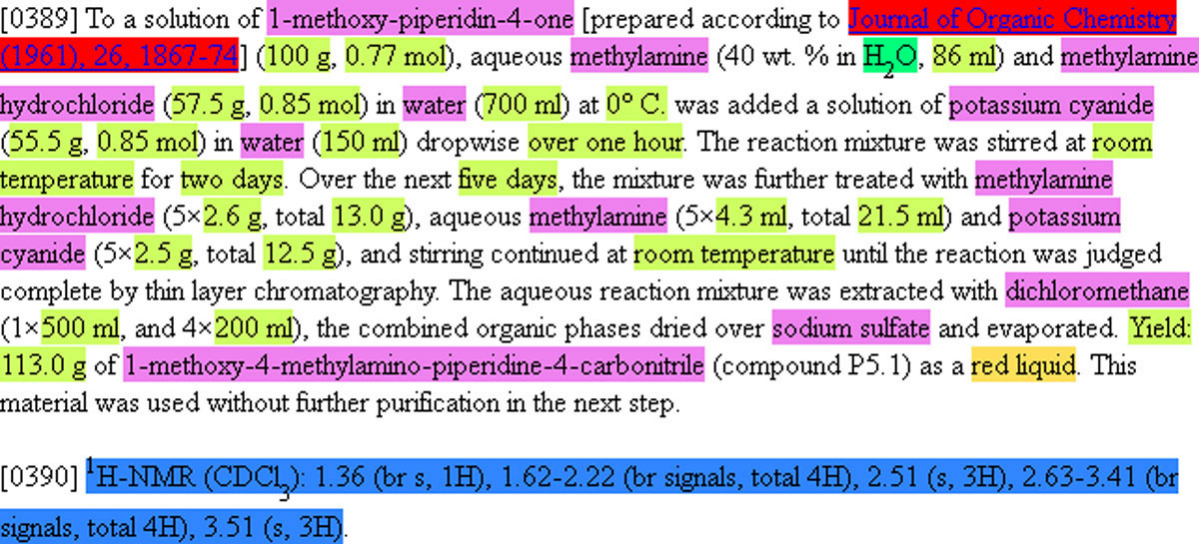
**Example of LeadMine annotation performed on a recent US patent**. Pink = molecule; red = journal reference; light green = physical quanity; turquoise = formula; orange = colorstate; blue = NMR

While this paper has focused on the chemical dictionaries and grammars used by our system, the underlying methodology of dictionaries, grammars, spelling correction and abbreviation detection are applicable to many other domains. For example we have produced grammars to recognize physical quantities, journal references and NMR spectra, and dictionaries for diseases and species (Figure 7).

## Conclusions

We have developed a fast high-precision solution to chemical entity recognition which differs from conventional machine learning approaches by being able to attribute all entities to a specific dictionary or grammar. As a result errors and omissions can be incrementally corrected. The use of dictionaries and grammars is not limited to chemistry allowing the system to recognize entities from other domains.

## Competing interests

The authors are employees of NextMove Software which sells a product (LeadMine) that contains the algorithms discussed in this paper.

## Authors' contributions

DL is the lead developer of LeadMine and evaluated its performance. RS assisted with algorithm development and dictionary/grammar preparation. DL drafted this manuscript with assistance from RS. Both authors read and approved the final manuscript.

## Supplementary Material

Additional file 1 The output of LeadMine, in CHEMDNER chemical entity mention annotation format, which was used to produce Figure 4. Click here for file

Additional file 2 The output of LeadMine, in CHEMDNER chemical entity mention annotation format, which was used to produce Figure 6. Click here for file

## Declarations

Funding for publication was provided by NextMove Software Ltd.

This article has been published as part of *Journal of Cheminformatics *Volume 7 Supplement 1, 2015: Text mining for chemistry and the CHEMDNER track. The full contents of the supplement are available online at http://www.jcheminf.com/supplements/7/S1.

## References

[B1] Krallinger M Rabal O Leitner F Vazquez M Salgado D Lu Z Leaman R Lu Y Ji D Lowe DM Sayle RA Batista-Navarro RT Rak R Huber T Rocktaschel T Matos S Campos D Tang B Xu H Munkhdalai T Ryu KH Ramanan SV Nathan S Zitnik S Bajec M Weber L Irmer M Akhondi SA Kors JA Xu S An X Sikdar UK Ekbal A Yoshioka M Dieb TM Choi M Verspoor K Khabsa M Giles CL Liu H Ravikumar KE Lamurias A Couto FM Dai H Tsai RT Ata C Can T Usie A Alves R Segura-Bedmar I Martinez P Oryzabal J Valencia A The CHEMDNER corpus of chemicals and drugs and its annotation principles J Cheminform 2015 7 Suppl 1 S2 10.1186/1758-2946-7-S1-S2 PMC4331692 25810773

[B2] Klinger R Kolarik C Fluck J Hofmann-Apitius M Friedrich CM Detection of IUPAC and IUPAC-like chemical names Bioinformatics 2008 24 i268 10.1093/bioinformatics/btn181 18586724 PMC2718657

[B3] Jessop DM Adams S Willighagen EL Hawizy L Murray-Rust P OSCAR4: a flexible architecture for chemical text-mining J Cheminformatics 2011 41 21999457 10.1186/1758-2946-3-41 PMC3205045

[B4] Rocktäschel T Weidlich M Leser U ChemSpot: a hybrid system for chemical named entity recognition Bioinformatics 2012 28 1633 1640 10.1093/bioinformatics/bts183 22500000

[B5] Vazquez M Krallinger M Leitner F Valencia A Text Mining for Drugs and Chemical Compounds: Methods, Tools and Applications Mol Inform 2011 30 506 519 10.1002/minf.201100005 27467152

[B6] Gurulingappa H Mudi A Toldo L Hofmann-Apitius M Bhate J Challenges in mining the literature for chemical information RSC Adv 2013 3 16194 16211 10.1039/c3ra40787j

[B7] Sayle R Xie PH Muresan S Improved Chemical Text Mining of Patents with Infinite Dictionaries and Automatic Spelling Correction J Chem Inf Model 2011 52 51 62 22148717 10.1021/ci200463r

[B8] Bolton EE Wang Y Thiessen PA Bryant SH Ralph A Wheeler and David C PubChem: Integrated Platform of Small Molecules and Biological Activities Annu Rep Comput Chem 2008 4 Spellmeyer Elsevier 217 241

[B9] Hettne KM Stierum RH Schuemie MJ Hendriksen PJ Schijvenaars BJ van Mulligen EM Kleinjans J Kors JA A Dictionary to Identify Small Molecules and Drugs in Free Text Bioinformatics 2009 19759196 10.1093/bioinformatics/btp535

[B10] Degtyarenko K de Matos P Ennis M Hastings J Zbinden M McNaught A Alcantara R Darsow M Guedj M Ashburner M ChEBI: a database and ontology for chemical entities of biological interest Nucleic Acids Res 2008 36 D344 350 17932057 10.1093/nar/gkm791 PMC2238832

[B11] Schwartz A Hearst M A Simple Algorithm for Identifying Abbreviation Definitions in Biomedical Text Proc Pac Symp Biocomput Kauai 2003 451 462 12603049

[B12] Lowe DM Sayle RA LeadMine: A grammar and dictionary driven approach to chemical entity recognition BioCreative Chall Eval Workshop. Washington 2013 2 47

